# Estrogen metabolism in the human lung: impact of tumorigenesis, smoke, sex and race/ethnicity

**DOI:** 10.18632/oncotarget.22269

**Published:** 2017-11-01

**Authors:** Jing Peng, Sibele I. Meireles, Xia Xu, William E. Smith, Michael J. Slifker, Stacy L. Riel, Shumenghui Zhai, Guo Zhang, Xiang Ma, Mindy S. Kurzer, Grace X. Ma, Margie L. Clapper

**Affiliations:** ^1^ Cancer Prevention and Control Program, Fox Chase Cancer Center, Philadelphia, PA 19111, USA; ^2^ Leidos Biomedical Research, Inc., Frederick, MD 21701, USA; ^3^ Department of Food Science and Nutrition and Department of Medicine, University of Minnesota, St. Paul, MN 55108, USA; ^4^ Center for Asian Health, Temple University, Philadelphia, PA 19140, USA

**Keywords:** estrogen metabolism, 4-hydroxy estrogen, non-small cell lung cancer, tobacco smoke, never-smoking Chinese women

## Abstract

Previous data from this group demonstrate that the murine lung metabolizes estrogen. Production of the putative carcinogen 4-hydroxyestrogen (4-OHE) is elevated within the lungs of female vs. male mice and accelerated by tobacco smoke. The goal of this study was to determine if the human lung metabolizes estrogen and evaluate the impact of tumor formation, smoke, sex and race/ethnicity on metabolism. Urine and lung tissue (normal, tumor) were obtained from 49 non-small cell lung cancer patients. Healthy postmenopausal Caucasian (*n =* 19) and Chinese (*n =* 20) American women (never-smokers) donated urine. Quantitative RT-PCR analyses indicate that multiple estrogen synthesis and metabolism genes are expressed in human bronchoalveolar cells. Estrogen and its metabolites were measured in lung tissue and urine using liquid chromatography/tandem mass spectrometry. Wilcoxon rank tests were used for statistical comparisons. E_1_, E_2_, E_3_ and estrogen metabolites 2-OHE_1_, 2-OHE_2_, 4-OHE_1_, 4-OHE_2_, 2-OME_1_ and 2-OME_2_ were detected at higher levels in tumor vs. adjacent normal tissue and in women vs. men (*P* < 0.05). The proportion of 4-OHEs was higher in tumors than in normal lung tissue (*P* < 0.05), and elevated in normal tissue from current- vs. never-smoking women (*P =* 0.006); similar trends were observed in urine. The proportion of 4-OHEs in the urine of postmenopausal Chinese American women was 1.8-fold higher than that of Caucasian women (*P* = 0.015). These data indicate that estrogen metabolites are present in the human lung. A shift towards 4-hydroxylation during lung tumorigenesis may contribute to the risk conferred by smoking, sex or race/ethnicity.

## INTRODUCTION

Smoking tobacco continues to be the primary risk factor for lung cancer development. However, the etiology of lung tumors that arise in never-smokers is much less clear (∼15% in men; > 50% in women worldwide) [1]. The percentage of never-smokers among women with lung cancer is much higher in Asia (60–80%) than in the US (15%) or Europe (20%) [2]. Even Chinese American women residing on the West coast of the US face a risk of lung cancer that is 4-fold higher than that of non-Hispanic American whites, after controlling for smoking status [3]. The basis for these sex and racial/ethnic differences in lung cancer incidence remains unknown.

Recent studies suggest that estrogen and progesterone play an important role in lung carcinogenesis [4]. While most studies have focused on receptor-mediated signaling, less attention has been given to the conversion of estrogen to oncogenic metabolites within the lung. In extrahepatic tissues, estrogens are hydroxylated by cytochrome P450 (CYP) 1A1 and 1B1 to form 2- and 4-hydroxylated estrogens (2-OHEs and 4-OHEs), respectively [5, 6]. 4-OHEs can bind and activate estrogen receptors (ERs) [6–8] and generate free radicals that cause DNA damage and oncogenic mutations [9]. 2-OHEs have weak estrogenic activity and are less reactive with DNA than 4-OHEs [7, 10, 11]. These catechol estrogens are conjugated via glucuronidation, sulfation and/or O-methylation for excretion [12]. Importantly, 2-methoxy estrogens (2-OMEs), generated by catechol-O-methyltransferase (COMT), are potent inhibitors of tumor cell proliferation and angiogenesis, and are thus protective [13, 14]. High levels of 4-OHEs [15] and less 2-hydroxylation of parent estrogens confer a higher risk of breast cancer [16]. Our data indicate that CYP1B1, the enzyme responsible for the production of 4-OHEs, contributes to the progression of head and neck cancers [17]. In addition, we found that estrogen was extensively metabolized in the mouse lung to form 2-OHEs, 4-OHEs and 2-OMEs [18]. Furthermore, the level of 4-OHE is higher in the lungs of female mice and elevated following tobacco smoke exposure [18], resulting from induction of *Cyp1b1* [19]. While several studies have shown that estrogen metabolism varies with smoking status [20] and race/ethnicity [21–25], the impact of these factors on estrogen metabolism in the human lung has not been investigated.

The goal of this study was to assess the ability of the human lung to metabolize estrogen and determine if metabolism is altered either during lung tumorigenesis or by tobacco smoke, sex or race/ethnicity. Elevated 4-hydroxylation of estrogen in lung tumors, smokers and Chinese American women suggests that 4-OHEs may be associated with lung cancer and sex/racial disparities. The results support further investigation of 4-OHEs as biomarkers of lung cancer risk, as well as targets for preventive intervention and treatment of lung cancer.

## RESULTS

### Expression of estrogen synthesis and metabolism genes in human bronchoalveolar cells

**Figure 1:**
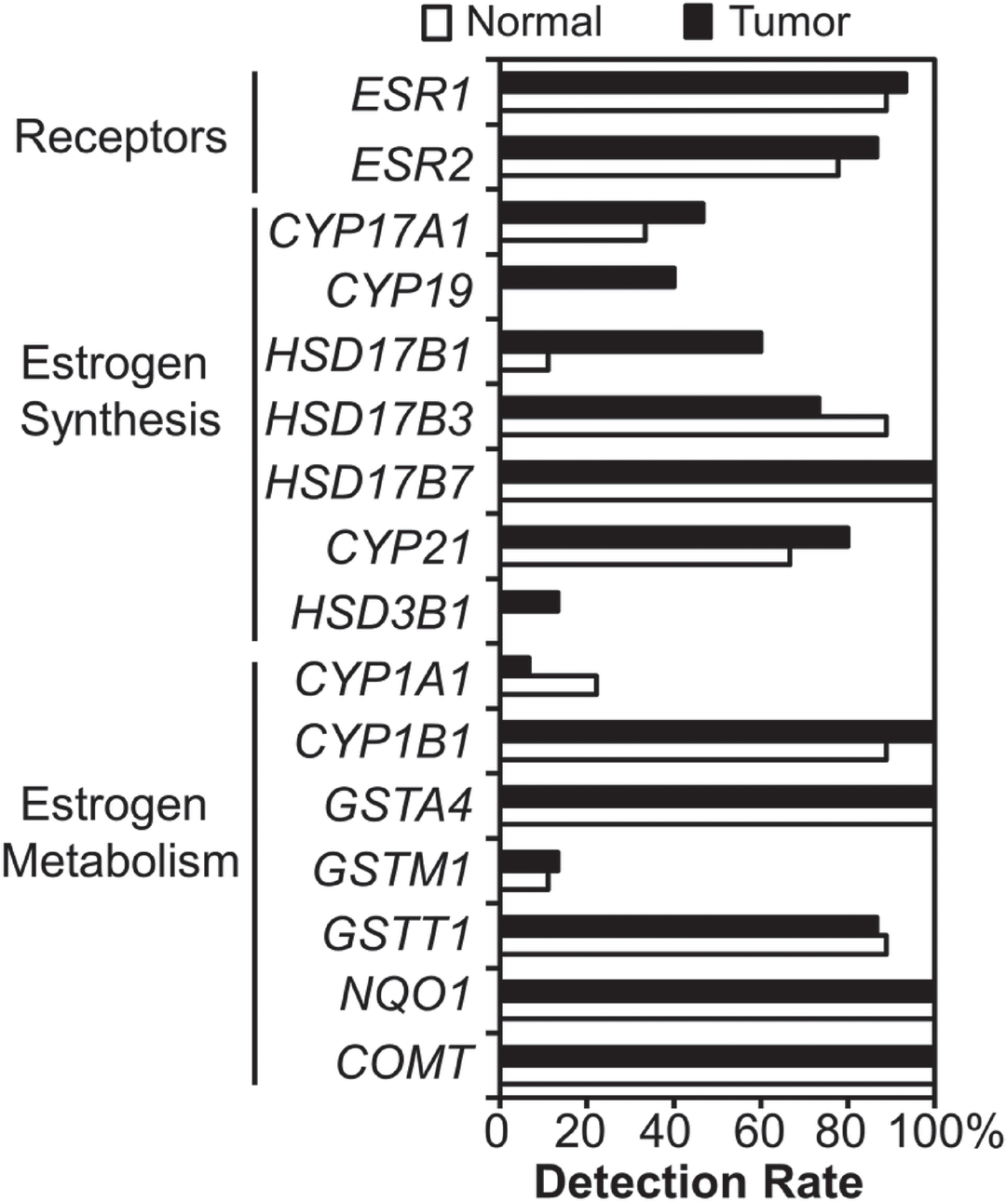
Expression of estrogen-related genes in human bronchoalveolar cells Cells were microdissected from the tumor and adjacent normal lung of NSCLC patients. RNA was extracted and analyzed by quantitative real-time RT-PCR. The percentage of samples with detectable transcripts (Ct values ≤ 35) is plotted for each gene.

The ability of the human lung to synthesize and metabolize estrogen was assessed by examining the level of expression of relevant genes in bronchoalveolar cells isolated from normal and tumor tissue (16 patients; age 53 – 81): 11 women, 5 men; 12 ever-smokers and 4 never-smokers. Of the tissues evaluated (14 normal and 9 tumor), 6 were matched pairs. Transcripts of all 14 metabolic genes evaluated (*CYP17A1, CYP19, HSD17B1, HSD17B3, HSD17B7, CYP21, HSD3B1, CYP1A1, CYP1B1, GSTA4, GSTM1, GSTT1, NQO1 and COMT*), as well as ER α and β, were detectable within the human lung (Figure 1). Interestingly, expression of several estrogen synthesis genes (*CYP17A, CYP19, HSD17B1, CYP21 and HSD3B1*) was detected more frequently in tumor tissue than in normal tissue, with *CYP19* (aromatase) and *HSD3B1* only detected in tumors. Genes involved in estrogen metabolism (*CYP1B1, GSTA4, GSTT1, NQO1 and COMT*) were also expressed at a high frequency in lung tissue (87–100%) (Figure 1), suggesting that human bronchoalveolar cells can both synthesize and metabolize estrogen.

### Estrogen and its metabolites are present in human lung tissue and altered during tumor formation

To confirm the function of estrogen metabolizing enzymes within the lung, tissue (tumor and adjacent normal) from 35 non-small cell lung cancer (NSCLC) patients was analyzed for 16 estrogens/estrogen metabolites using a liquid chromatography/tandem mass spectrometry (LC-MS^2^) assay. Eighty percent of all subjects were non-Hispanic whites and 89% of the women were postmenopausal (16 ≥ age 55 plus one hysterectomy). E_1_, E_2_, E_3_ and 6 estrogen metabolites (2-OHE_1_, 2-OHE_2_, 4-OHE_1_, 4-OHE_2_, 2-OME_1_, and 2-OME_2_) were detected within the human lung (Table 1), while 4-methoxyestrone (4-OME_1_), 4-methoxyestradiol (4-OME_2_), 2-hydroxyestrone-3-methyl ether (3-OME_1_), 16α-hydroxyestrone (16α-OHE_1_), 16-epiestriol (16-epiE_3_), 17-epiestriol (17-epiE_3_), and 16-ketoestradiol (16-ketoE_2_) were not detected. E_2_ was the most abundant pulmonary estrogen species detected and 2-OHE_1_ was the estrogen metabolite present at the highest concentration in the human lung (Table 1). Concentrations of 4-OHEs were lower than those of 2-OHEs and 2-OMEs. Interestingly, although the concentration of E_2_ in human lung tissue was higher than that of E_1_, levels of 2-OHE_2_, 2-OME_2_ and 4-OHE_2_ were lower than those of 2-OHE_1_, 2-OME_1_ and 4-OHE_1_.

**Table 1:** Levels of estrogen and its metabolites in lung tissue*

NORMAL	All (pg/g tissue)				Never Smokers (pg/g tissue)				Current Smokers (pg/g tissue)			
	W *n* = 18	M *n* = 17	*P* W vs. M	FDR	W *n* = 9	M *n* = 7	*P* W vs. M	FDR	W *n* = 9	M *n* = 10	*P* W vs. M	FDR
E_1_	8.48	6.10	**0.010**	**0.013**	6.85	5.47	0.091	0.131	10.32	6.46	**0.035**	**0.038**
E_2_	15.12	8.98	**4.3x10**^-8^	**1.9x10**^-7^	13.11	8.73	**1.8x10**^-4^	**0.001**	18.19	9.42	**8.6x10**^-5^	**1.9x10**^-4^
E_3_	3.53	2.57	**0.003**	**0.004**	3.02	2.14	0.210	0.249	4.93	2.67	**0.003**	**0.004**
2OHE_1_	2.69	1.86	**3.2x10**^-6^	**6.9x10**^***-6***^	2.32	1.69	**0.003**	**0.007**	2.97	1.98	**8.7x10**^-5^	**1.9x10**^-4^
2OHE_2_	1.28	0.71	**8.6x10**^-8^	**2.8x10**^-7^	1.04	0.72	**0.002**	**0.007**	2.16	0.68	**4.3x10**^-5^	**1.4x10**^-4^
4OHE_1_	0.39	0.21	**1.8x10**^-5^	**3.3x10**^-5^	0.30	0.20	0.031	0.058	0.45	0.21	**2.2x10**^-5^	**1.4x10**^-4^
4OHE_2_	0.16	0.10	**0.003**	**0.004**	0.12	0.10	0.457	0.457	0.35	0.10	**4.3x10**^-5^	**1.4x10**^-4^
2OME_1_	1.47	0.51	**3.1x10**^-9^	**4.0x10**^-8^	1.38	0.51	**3.5x10**^-4^	**0.002**	1.55	0.52	**4.3x10**^-5^	**1.4x10**^-4^
2OME_2_	0.88	0.26	**8.4x10**^-9^	**5.4x10**^-8^	0.85	0.25	**1.8 x10**^-4^	**0.001**	0.92	0.30	**1.5x10**^-4^	**2.8x10**^-4^
2OHEs/Total	0.11	0.12	0.883	0.883	0.10	0.12	0.351	0.380	0.12	0.12	0.356	0.356
4OHEs/Total	0.02	0.01	0.732	0.793	0.01	0.01	0.042	0.067	0.02	0.01	**0.004**	**0.005**
2OMEs/Total	0.07	0.04	**7.0x10**^-7^	**1.8x10**^-6^	0.06	0.04	**0.003**	**0.007**	0.09	0.04	**4.1x10**^-4^	**6.7x10**^-4^
4OHEs/2OHEs	0.13	0.12	0.503	0.595	0.11	0.12	0.142	0.184	0.17	0.12	**0.013**	**0.016**

TUMOR	W *n* = 17	M *n* = 15	*P* W vs. M	FDR	W *n* = 8	M *n* = 5	*P* W vs. M	FDR	W *n* = 9	M *n* = 10	*P* W vs. M	FDR
E_1_	9.79	5.84	**4.9x10**^-7^	**1.1x10**^-6^	8.77	5.20	**0.002**	**0.003**	12.02	6.79	**2.2x10**^-5^	**7.0x10**^-5^
E_2_	20.86	9.18	**1.4 x10**^-8^	**6.3x10**^-8^	18.73	8.96	**0.002**	**0.003**	24.36	10.15	**2.2x10**^-5^	**7.0x10**^-5^
E_3_	4.87	2.88	**1.8x10**^-6^	**2.9x10**^-6^	4.22	2.26	**0.011**	**0.016**	5.18	2.90	**1.5x10**^-4^	**2.2x10**^-4^
2OHE_1_	4.10	1.96	**4.9x10**^-7^	**1.1x10**^-6^	3.43	1.92	**0.002**	**0.003**	4.76	2.09	**4.3x10**^-5^	**8.0x10**^-5^
2OHE_2_	1.82	0.76	**7.1x10**^-9^	**4.6x10**^-8^	1.23	0.76	**0.002**	**0.003**	2.47	0.74	**2.2x10**^-5^	**7.0x10**^-5^
4OHE_1_	0.70	0.23	**3.2x10**^-6^	**4.7x10**^-6^	0.69	0.23	**0.011**	**0.016**	0.70	0.24	**8.7x10**^-5^	**1.4x10**^-4^
4OHE_2_	0.31	0.12	**9.5x10**^-6^	**1.2x10**^-5^	0.17	0.13	0.065	0.085	0.42	0.12	**2.2x10**^-5^	**7.0x10**^-5^
2OME_1_	1.73	0.62	**7.1x10**^-9^	**4.6x10**^-8^	1.73	0.49	**0.002**	**0.003**	1.73	0.63	**4.3x10**^-5^	**8.0x10**^-5^
2OME_2_	1.13	0.32	**2.5x10**^-8^	**8.0x10**^-8^	1.37	0.27	**0.002**	**0.003**	1.13	0.34	**4.3x10**^-5^	**8.0x10**^-5^
2OHEs/Total	0.12	0.12	0.411	0.411	0.12	0.12	0.222	0.262	0.11	0.11	1.000	1.000
4OHEs/Total	0.02	0.02	0.261	0.283	0.02	0.02	0.943	0.943	0.02	0.02	0.095	0.103
2OMEs/Total	0.06	0.04	**1.8x10**^-6^	**2.9x10**^-6^	0.07	0.04	**0.006**	**0.012**	0.06	0.04	**0.001**	**0.001**
4OHEs/2OHEs	0.14	0.13	0.165	0.194	0.14	0.13	0.943	0.943	0.21	0.13	**0.028**	**0.033**

^*^Values represent the medians. Estrogen and its metabolites in lung tissue from NSCLC patients were analyzed by LC-MS^2^. Statistical comparisons were based on the Wilcoxon rank sum test, with *P* ≤ 0.05 considered significant (underlined). Bold highlight denotes where both the *P* values and FDR are ≤ 0.05. W: women; M: men.

**Figure 2:**
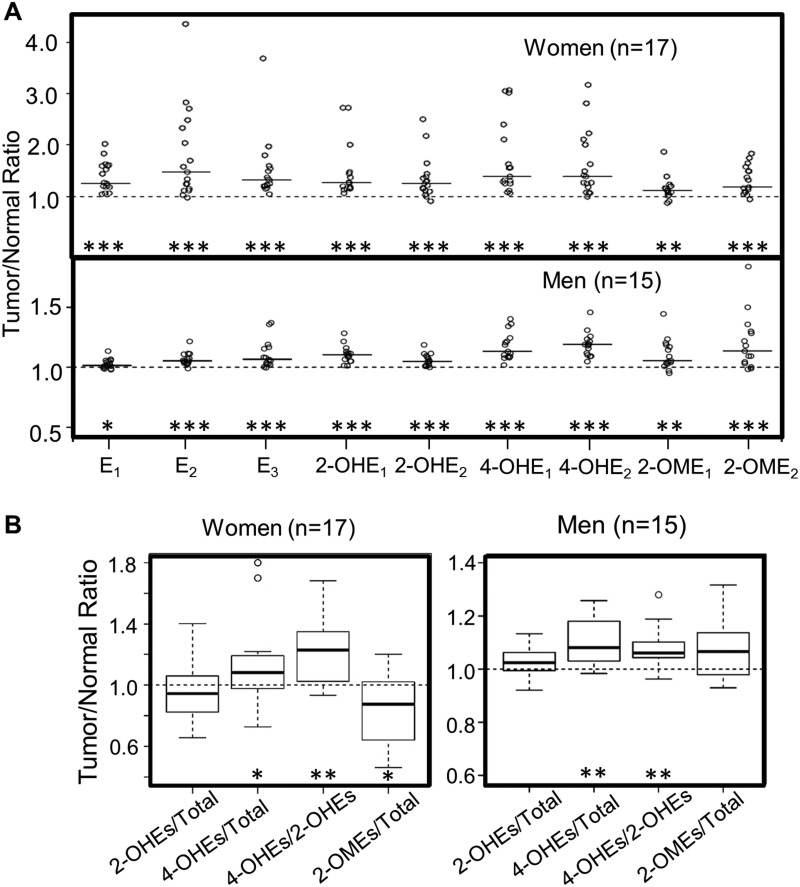
Comparison of estrogen and estrogen metabolites in lung tumors vs. adjacent normal lung tissue from NSCLC patients (**A**) Fold difference (tumor/normal ratio) in estrogen and estrogen metabolites in tumor vs. paired normal lung tissue. The median of the independent values (open circles) is shown as a solid horizontal line. (**B**) The level of 2-OHEs, 4-OHEs and 2-OMEs (% total estrogens and estrogen metabolites) and the ratio of 4-OHEs/2-OHEs in lung tumors vs. paired normal lung tissue were calculated. The fold difference between tumor and paired normal lung tissue (tumor/normal ratio) is plotted. The box plots denote the median and the 75th and 25th percentiles (inter-quartile range, IQR). The “whiskers” represent the most extreme points (≤ 1.5 times the IQR). Outliers are plotted outside the box. Values above 1 indicate the levels of estrogen or estrogen metabolites are higher in lung tumors. Comparisons between normal and tumor tissue were performed using the Wilcoxon signed rank test. The FDR was calculated to adjust for multiple comparisons (^*^P < 0.05; ^**^P < 0.01; ^***^P < 0.001).

To assess if estrogen synthesis and metabolism are altered during lung tumorigenesis, the levels of estrogen and its metabolites in lung tumors were compared to those of matched normal tissue (paired samples were available for 32 of the 35 NSCLC patients). The level of each estrogen and estrogen metabolite was higher in the tumor as compared to matched normal tissue (*P* < 0.05, Figure 2A), irrespective of the patient’s sex and smoking status. Most importantly, the percentage of 4-OHEs remained significantly elevated in lung tumors vs. matched normal tissue from both men and women (*P* < 0.05), while the percentage of 2-OHEs did not differ significantly between lung tumors and normal tissue in women (*P* = 0.21) or men (*P* = 0.12) (Figure 2B). As a result, the ratio of 4-OHEs/2-OHEs was significantly higher in lung tumors as compared to paired normal tissue (*P* < 0.001, Figure 2B). Interestingly, the proportion of protective 2-OMEs was lower in lung tumors than in normal tissue from women (*P* = 0.014), but a trend of higher levels in tumor vs. normal tissue was observed in men (*P* = 0.08) (Figure 2B).

### Sex differences in the level of estrogen and estrogen metabolites in human lung tissue

**Figure 3:**
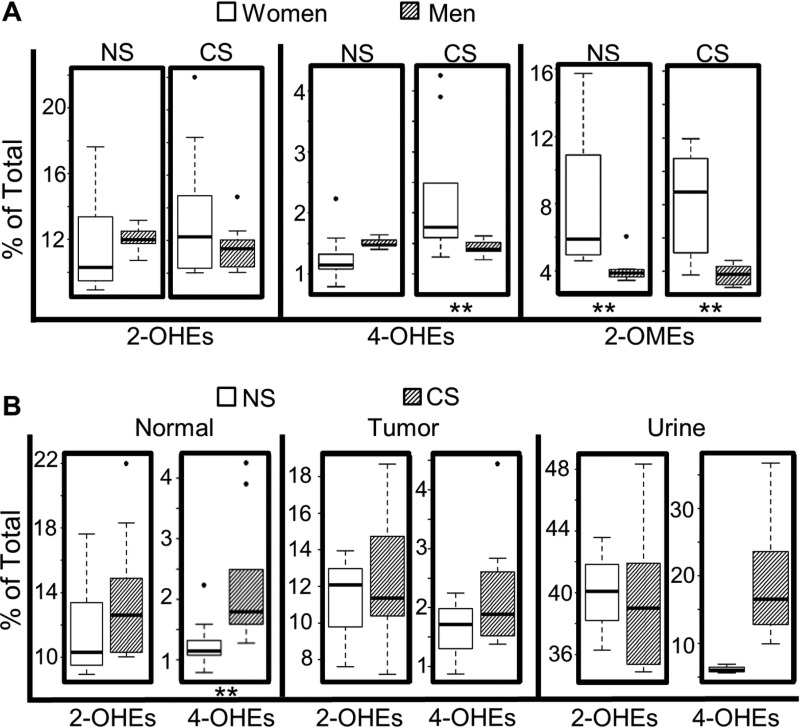
Impact of sex and smoking status on levels of estrogen metabolites (**A**) Comparison of estrogen metabolites in normal lung tissue from women vs. men. (**B**) Comparison of 4-OHEs and 2-OHEs in normal lung tissue, lung tumors and urine from women with NSCLC who are never-smokers (NS) or current-smokers (CS). Box plots denote the median and the 75th and 25th percentiles (inter-quartile range, IQR). The “whiskers” represent the most extreme points (≤ 1.5 times the IQR). Outliers are outside the box. *P* values are based on the Wilcoxon rank sum test (^**^P < 0.01).

As expected, the levels of most estrogens and estrogen metabolites, including E_2_, 2-OHE_1_, 2-OHE_2_, 4-OHE_1_, 2-OME_1_, and 2-OME_2_, were significantly higher in normal lung tissue from women vs. men who never smoked. Likewise, the levels of all estrogen metabolites, except for 4-OHE_2_, were higher in tumors from never-smoking women as compared to never-smoking men. Among current-smokers, the levels of all 9 estrogens and estrogen metabolites detected were elevated significantly in both normal lung tissue and tumors from women vs. men (Table 1). Interestingly, the percentage of 4-OHEs ((4-OHE_1_ + 4-OHE_2_)/total) within the normal lung was lower in women vs. men who never-smoked, while the opposite was observed among current-smokers (Figure 3A). In contrast, no significant sex difference in the percentage of 2-OHEs was observed in human lung tissue (normal or tumor), irrespective of smoking status. The percentage of 2-OMEs was ∼2-fold higher in both normal lung tissue and tumors from women as compared to men, independent of smoking history.

### Impact of tobacco smoke on estrogen metabolism within the human lung

To elucidate the effect of tobacco smoke on estrogen metabolism, the concentration of estrogens and estrogen metabolites in lung tissue (normal or tumor) from NSCLC patients who never smoked was compared with that of current-smokers. Among women, smoking significantly increased the level of 2-OHE_2_, 4-OHE_1_ and 4-OHE_2_ within the normal lung, and 2-OHE_2_ and 4-OHE_2_ in lung tumors, but did not alter the level of 2-OMEs (2-OME_1_ or 2-OME_2_) in either normal or tumor tissue (Table 2). Furthermore, both the percentage of 4-OHEs and the ratio of 4-OHEs/2-OHEs were increased significantly in normal lung tissue from current- vs. never-smoking women (Table 2, Figure 3B), but not in corresponding tumor tissue (Figure 3B). After adjusting for multiple comparisons, the difference in 2-OHE_2_, 4-OHE_2_, 4-OHEs/total and 4-OHEs/2-OHEs between current- and never-smoking women was still significant with a false discovery rate (FDR) < 0.05. Interestingly, smoking did not cause any significant change in the level of pulmonary estrogen metabolites in men with NSCLC (Table 3). For those women with NSCLC where matched samples of urine and lung tissue were available, the percentage of urinary 4-OHEs was higher in current- vs. never-smokers (Figure 3B). However, the increase did not achieve statistical significance, most likely due to the limited sample size.

**Table 2:** Levels of estrogen and its metabolites in never- vs. current-smoking women*

	Normal (pg/g tissue)				Tumor (pg/g tissue)				Urine (pg/mg creatinine)			
	N *n =* 9	C *n =* 9	*P* N vs. C	FDR	N *n =* 8	C *n =* 9	*P* N vs. C	FDR	N *n =* 3	C *n =* 4	*P* N vs. C	FDR
E_1_	6.85	10.32	0.340	0.402	8.77	12.02	0.074	0.242	24.97	32.70	0.857	1.000
E_2_	13.11	18.19	0.190	0.275	18.73	24.36	0.200	0.371	8.24	12.75	0.857	1.000
E_3_	3.02	4.93	0.077	0.167	4.22	5.18	0.200	0.371	20.85	28.02	1.000	1.000
2OHE_1_	2.32	2.97	0.094	0.174	3.43	4.76	0.139	0.361	32.42	60.74	1.000	1.000
2OHE_2_	1.04	2.16	**0.004**	**0.017**	1.23	2.47	0.021	0.234	13.56	43.25	0.628	1.000
4OHE_1_	0.30	0.45	0.050	0.131	0.69	0.70	0.321	0.418	5.57	22.11	0.114	0.743
4OHE_2_	0.12	0.35	**0.001**	**0.008**	0.17	0.42	0.036	0.234	1.94	11.58	0.057	0.495
2OME_1_	1.38	1.55	0.297	0.386	1.73	1.73	0.815	0.883	7.64	11.05	0.400	1.000
2OME_2_	0.85	0.92	0.387	0.419	1.37	1.13	0.673	0.795	1.89	2.59	0.400	1.000
2OHEs/Total	0.10	0.12	0.162	0.262	0.12	0.11	1.000	1.000	0.40	0.37	0.400	1.000
4OHEs/Total	0.01	0.02	**0.006**	**0.018**	0.02	0.02	0.277	0.400	0.06	0.18	0.057	0.371
2OMEs/Total	0.06	0.09	1.000	1.000	0.07	0.06	0.236	0.383	0.08	0.07	1.000	1.000
4OHEs/2OHEs	0.11	0.17	**0.001**	**0.008**	0.14	0.21	0.059	0.242	0.15	0.52	0.057	0.371

^*^Values represent the medians. Statistical comparisons were based on the Wilcoxon rank sum test, with *P* ≤ 0.05 considered significant (underlines above as indicated). Bold highlight denotes FDR values ≤ 0.05. N: never smokers; C: current smokers.

**Table 3:** Levels of estrogen and its metabolites in the lung of never- vs. current-smoking men*

	Normal (pg/g tissue)			Tumor (pg/g tissue)		
	N *n =* 7	C *n =* 10	*P* N vs. C	N *n =* 5	C *n =* 10	*P* N vs. C
E_1_	5.47	6.46	0.23	5.20	6.79	0.08
E_2_	8.73	9.42	0.19	8.96	10.15	0.17
E_3_	2.14	2.67	0.81	2.26	2.90	0.59
2OHE_1_	1.69	1.98	0.42	1.92	2.09	0.68
2OHE_2_	0.72	0.68	0.81	0.76	0.74	0.86
4OHE_1_	0.20	0.21	0.54	0.23	0.24	0.77
4OHE_2_	0.10	0.10	0.81	0.13	0.12	0.95
2OME_1_	0.51	0.52	0.89	0.49	0.63	0.77
2OME_2_	0.25	0.30	0.60	0.27	0.34	0.25
2OHEs/Total	0.12	0.12	0.19	0.12	0.11	0.25
4OHEs/Total	0.01	0.01	0.11	0.02	0.02	0.13
2OMEs/Total	0.04	0.04	0.74	0.04	0.04	0.77
4OHEs/2OHEs	0.12	0.12	0.89	0.14	0.13	0.95

^*^Values represent the medians. Statistical comparisons were based on the Wilcoxon rank sum test. N: never smokers; C: current smokers.

### Differences in the urinary estrogen metabolites of Caucasian and Chinese American women

**Figure 4:**
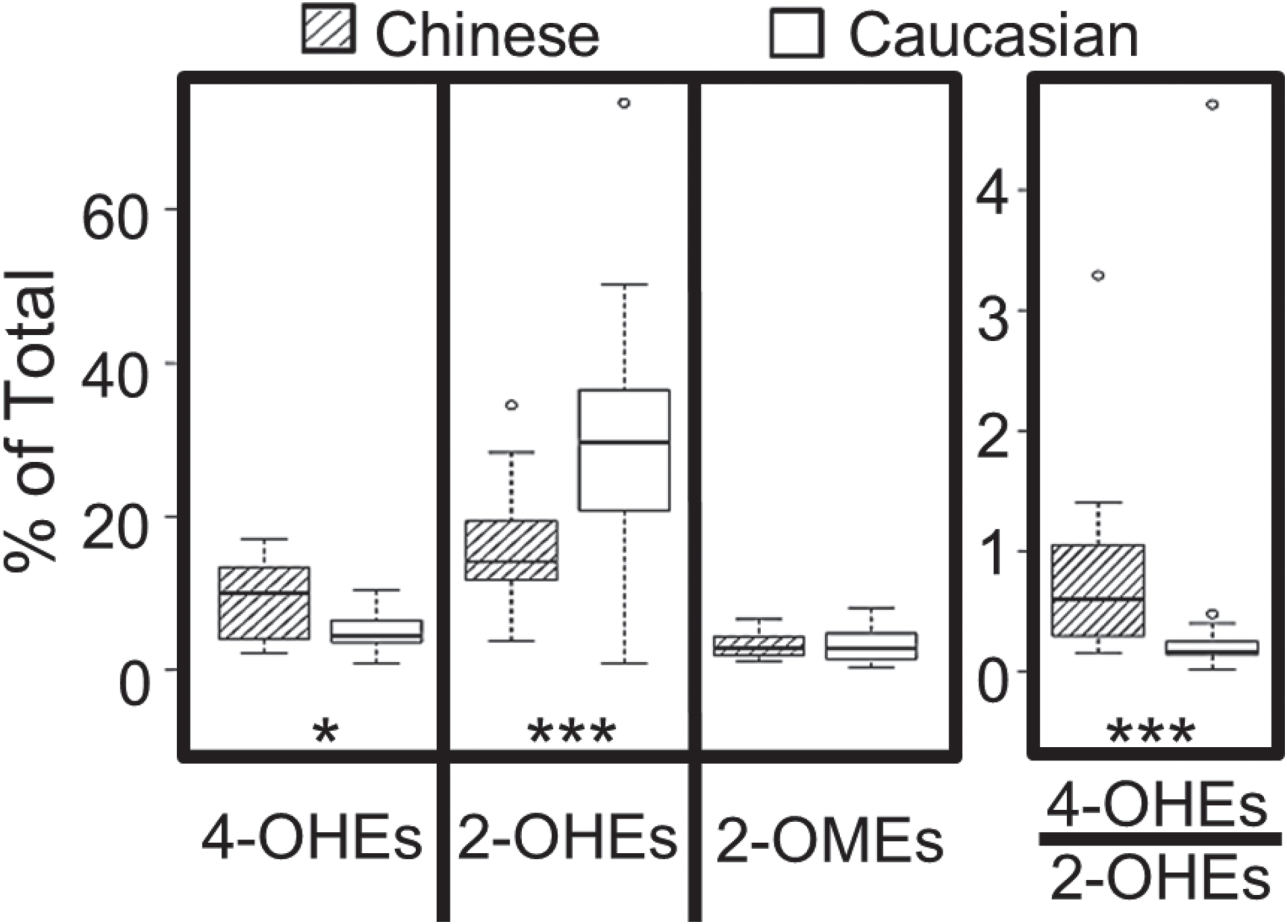
Level of urinary catechol estrogens (% total estrogens and estrogen metabolites) in healthy postmenopausal Chinese (*n* = 20) vs. Caucasian (*n* = 19) American women who never smoked The box plots denote the median and the 25th and 75th percentiles (inter-quartile range, IQR). The “whiskers” represent the most extreme points (≤ 1.5 times the IQR). Outliers are outside the boxes. *P* values are based on the Wilcoxon rank sum test (^*^P < 0.05; ^***^P < 0.001).

Potential racial differences in estrogen metabolism were explored by comparing the levels of urinary estrogens and estrogen metabolites from postmenopausal Caucasian and Chinese American women who never smoked. All Chinese American women donors were healthy and residing in Philadelphia. The majority (89%) were immigrants, with a mean time in the US of 19 years. Levels of urinary estrogens and estrogen metabolites from Chinese American women (*N* = 20, age 55–65) were compared with archival data for 19 healthy Caucasian women matched for age and body mass index (19.2–24.8). As shown in Figure 4, Chinese American women had 80% more 4-OHEs (% total) (*P* = 0.015) and 2-fold less 2-OHEs (% total) (*P* = 0.0002) in their urine as compared to Caucasian women. The percentage of 2-OMEs did not differ significantly with race/ethnicity. The ratio of urinary 4-OHEs/2-OHEs was also 80% higher (*P* = 0.0001) in Chinese women.

## DISCUSSION

The present study is the first to demonstrate that estrogen metabolites are present in human lung tissue. This finding extends previous reports from others indicating that E_1_ and E_2_ and transcripts/proteins encoded by estrogen synthesis genes [31–33] are present in human lung tissue. Gene expression profiling of bronchoalveolar cells revealed that the lung could both synthesize and metabolize estrogens. Detection of unconjugated 2-OHEs, 4-OHEs, 2-OMEs, but not 16-OHEs (16α-OHE_1_, 16-epiE_3_, 17-epiE_3_, 16-ketoE_2_) suggests that pulmonary estrogens are metabolized primarily via 2- and 4-hydroxylation. Of note, 16-hydroxy estrogens were not detected in the murine lung after hydrolysis, thus it is unlikely that 16α-OHE_1_ is involved in lung carcinogenesis. Because the human lung tissue was not perfused, one cannot conclude definitively that the human lung metabolizes estrogen. However, while unconjugated 2-OHE and 4-OHE were detected in human lung tissue in the present study, 2-OHE or 4-OHE have not been detected previously in serum in unconjugated forms [16, 27]. This difference suggests that some estrogen metabolites are produced locally in the lung.

Consistent with enhanced estrogen synthesis in lung tumors [31, 34], our results demonstrate that estrogen/estrogen metabolite levels are elevated in lung tumors as compared to paired normal tissue (Figure 2). However, the observed elevation of 4-OHEs (% total) and 4-OHEs/2-OHEs in lung tumors suggests a shift in metabolism towards 4-hydroxylation during tumor formation. Given that 4-OHEs are estrogenic and genotoxic, an increase could promote tumor development. Interestingly, the percentage of protective estrogen metabolites (2-OMEs) is reduced in lung tumors from women, but increased, although not significantly, in tumors from men. This suggests a sex difference in the O-methylation of catechol estrogens during lung tumor formation. Future longitudinal and/or case-control studies are required to fully understand the relationship between estrogen metabolism and lung cancer and determine if high levels of oncogenic estrogen metabolites and/or low levels of protective estrogen metabolites accelerate lung carcinogenesis.

Data from the present study provide strong evidence that estrogen metabolism varies with sex, smoking status and race/ethnicity, all risk factors for lung cancer. Higher levels of both pulmonary estrogens and estrogen metabolites in women vs. men could lead to enhanced activation of ER signaling in women and accelerate tumor progression. The level of E_2_ within the lungs (normal and tumor tissue) of Japanese postmenopausal women with NSCLC is lower than that of men [34], a discrepancy that may be attributed to differences in smoking status (not reported) or race/ethnicity. In the present study, the sex difference in the percentage of 4-OHEs depended on smoking status: 4-OHEs were more abundant in normal lung tissue of never-smoking men vs. women. The opposite was observed among current-smokers (Figure 3A). Because smoking shifts estrogen metabolism towards 4-hydroxylation in women but not in men (Figure 3B), the percentage of 4-OHEs in the normal lung tissue of women may be elevated to the same level as in men after smoke exposure. Sex differences were also observed in the levels of protective 2-OMEs (both as absolute concentration and % total), which were higher in the lungs (normal and tumor tissue) of women as compared to men, irrespective of smoking status.

This is the first report that tobacco smoke alters pulmonary estrogen metabolites in humans. Tobacco smoke did not induce a significant change in the level of any of the estrogen metabolites within the lungs of men (normal or tumor, Table 3), but significantly changed estrogen metabolism within the lungs of women. Tobacco smoke exposure led to increased levels of 2-OHE_2_ (*P* = 0.004) in normal lung tissue, although it did not alter the percentage of 2-OHEs or the levels of 2-OMEs (Table 2). However, 4-hydroxylation (4-OHE_1_ and 4-OHE_2_ levels, the percentage of 4-OHEs and the ratio of 4-OHEs/2-OHEs) in normal lung tissue was increased significantly among current-smokers as compared to never-smokers. Therefore, tobacco smoke induces a shift in estrogen metabolism towards a harmful pathway, which could contribute to lung carcinogenesis specifically in women. On the other hand, neither the percentage of 4-OHEs nor the 4-OHEs/2-OHEs ratio was different in lung tumors from current- and never-smokers, suggesting that other factors increase 4-hydroxylation of estrogens in never-smokers during carcinogenesis. Furthermore, tobacco smoke exposure elevated 4-OHEs (% total) in both lung tissue and matched urine samples (the urinary results did not achieve significance, possibly due to the small sample size). This finding is consistent with that of a recent study showing that tobacco smoke increased 2- and 4-hydroxylation and decreased O-methylation of estrogens in the urine of healthy pre-menopausal women [20]. It also suggests that the smoking-induced increase in 4-hydroxylation of estrogen in the lung is present in urine samples. Additional studies are needed to confirm the potential use of urine as a surrogate for estrogen metabolite levels in lung tissue.

Data from this study indicate that never-smoking postmenopausal Chinese women have a more active 4-hydroxylation pathway (and less 2-hydroxylation activity) than Caucasians. Interestingly, the levels of 2-OMEs (% total) did not differ between the two groups (Figure 4). Racial/ethnic differences in estrogen metabolism could result from race/ethnic group-specific polymorphisms in estrogen metabolizing genes [21] as well as environmental exposures due to varying life styles (i.e. soy (phytoestrogen) intake [35], exposure to cooking oil fumes and/or secondhand smoke). A larger case controlled study is needed to assess the contribution of excess 4-OHEs to the high risk of lung cancer faced by never-smoking Chinese women. Given the genotoxicity of 4-OHEs and the high frequency of acquired EGFR mutations in Asian women with lung cancer [36], it will be important to determine if 4-OHEs accelerate the rate of EGFR mutation.

Striking similarity exists between the metabolism of estrogen in the lungs of mice [18] and humans. First, E_1_, E_2_, E_3_, 2-OHEs, 4-OHEs and 2-OMEs, but not 16-hydroxy estrogens, were detected in both species, with E_2_ being the most abundant. However, although 4-OHEs were the most abundant estrogen metabolites in the murine lung, their level was less than that of 2-OHEs and 2-OMEs in the human lung. Second, the level of most estrogens and estrogen metabolites was higher in females (mice and humans). Third, exposure to tobacco smoke increased the production of 4-OHEs. The impact of over production of this putative carcinogen on lung cancer risk is of great interest.

In summary, the discovery of estrogen metabolism within the human lung provides exciting opportunities to further investigate the role of estrogen metabolites in lung tumorigenesis. The present data demonstrate a higher proportion of 4-OHEs and a shift from 2-hydroxylation to 4-hydroxylation (higher 4-OHEs/2-OHEs ratio) in lung tumors (vs. paired normal tissue) and in urine samples from populations at higher risk for lung cancer, including current-smokers (vs. never-smokers) and never-smoking Chinese American women (vs. Caucasian women). Future studies will assess the utility of 4-OHEs (in tissue and/or urine) as biomarkers of lung cancer risk. Therapeutic interventions that either inhibit the production of 4-OHEs and/or promote the generation of 2-OMEs could confer protection from genotoxicity. Establishment of a link between the causal effects of 4-OHEs and lung cancer will provide strong rationale for targeting the enzyme(s) responsible for their synthesis, a novel strategy for the prevention of this disease.

## MATERIALS AND METHODS

### Subjects and specimens

NSCLC patients undergoing surgery (1998–2014) at Fox Chase Cancer Center (FCCC), were enrolled irrespective of sex, age, race/ethnicity, stage, prior chemotherapy or radiation, smoking history or menopausal status. Lung tissue (paired normal-appearing and tumor) and/or urine samples were collected at the time of surgery for gene expression analyses and/or measurement of estrogens and estrogen metabolites. Never-smokers were defined as people who used < 100 cigarettes in their lifetime. Individuals were categorized as former- or current-smokers based on quitting smoking ≥ or < 6 months prior to surgery, respectively. Table 4 summarizes the characteristics of all NSCLC patients. Detailed information for each subject is provided in Supplementary Table 1.

**Table 4:** Characteristics of subjects with non-small cell lung cancer

	NSCLC Patients *N* = 49 (% total)
Race/Ethnicity Caucasian	39 (80%)
African American Asian	7 (14%) 3 (6%)
Gender Male	22 (45%)
Female	27 (55%)
Age Range: 38 – 81 ≥ 55 < 55	43 (88%) 6 (12%)
Smoking Status^*^ Never-Smokers	18 (37%)
Ever-Smokers	31 (63%)
Current Former Quitting Time Unknown	19 (39%) 8 (16%) 4 (8%)
Menopause status (27 women) Post-menopausal^**^ Unknown	25 (93%) 2 (7%)
Histopathology Adenocarcinoma Squamous Cell Carcinoma Mixed Adeno and Squamous	44 (90%) 4 (8%) 1 (2%)
Lung Tissue Sample Tumor Normal Paired	44 (90%) 43 (88%) 32 (65%)
Urine Sample	15 (31%)

^*^ Never smokers - defined as individuals who had smoked fewer than 100 cigarettes in their lifetime. Individuals with a smoking history were categorized as former or current smokers based on when they quit smoking (≥ or < 6 months prior to surgery, respectively).

^**^ Includes 24 women ≥ 55 years of age and one woman (47 years old) who underwent hysterectomy (ovaries intact).

Self-identified healthy Chinese American women were recruited from the Philadelphia area. A one-time urine sample was collected from eligible, postmenopausal never-smokers and shipped to the University of Minnesota for estrogen analyses. The urinary estrogen metabolite profiles of Asian women were compared to archived samples from Caucasian American women matched for smoking status and of a similar age and body mass index. All studies were approved by the Institutional Review Boards at FCCC, Temple University and the University of Minnesota.

### Gene expression analyses

Surgically resected lung tissue was snap-frozen in optimal cutting temperature medium. Bronchoalveolar cells (∼1500, normal and tumor) were laser microdissected from H&E stained sections (5 µm) using the PixCell® IITM system (Arcturus Bioscience, Inc.). RNA was isolated and qRT-PCR performed [26] using gene-specific primers (Table 5).

**Table 5:** qRT-PCR gene-specific primers (Life Technologies)

Genes	Primer Catalogue Numbers
*TFRC*	Hs99999911_m1
*TBP*	Hs99999910_m1
*18S*	Hs99999903_m1
*ESR1*	Hs00174860_m1
*ESR2*	Hs00230957_m1
*CYP11B1*	Hs01596404_m1
*CYP17A1*	Hs00164375_m1
*CYP21*	Hs00416901_g1
*CYP19*	Hs00903406_m1
*HSD3B1*	Hs00426435_m1
*HSD17B1*	Hs00166219_g1
*HSD17B3*	Hs00609319_m1
*HSD17B7*	Hs00996127_m1
*CYP1B1*	Hs00164383_m1
*CYP1A1*	Hs00153120_m1
*GSTM1*	Hs02341469_m1
*GSTA4*	Hs00155308_m1
*GSTT1*	Hs00184475_m1
*NQO1*	Hs00168547_m1
*COMT*	Hs00241349_m1

### Measurement of estrogens and estrogen metabolites in lung tissue

Profiling of estrogen and estrogen metabolites in lung tissue was performed at Leidos Biomedical Research, Inc., Frederick, MD, using a LC-MS^2^ assay described previously by this group [18], except that samples were not hydrolyzed to remove β-glucuronide and sulfate conjugates from estrogen metabolites. The limit of detection was approximately 20 fg estrogen metabolite on column. The same protocol, when used to extract estrogen metabolites from another highly complex protein mixture (serum), yielded an extraction efficiency of 90–105%.

### Measurement of estrogens and estrogen metabolites in urine

Estrogens and estrogen metabolites were extracted from urine samples of NSCLC patients and analyzed at Leidos Biomedical Research, Inc., Frederick (without hydrolysis). Urine samples from healthy Caucasian and Chinese American women were processed and analyzed at the University of Minnesota, as described previously [28]. Estrogens and estrogen metabolites (12 species, excluding 16-epiE_3_, 17-epiE_3_, 16-ketoE_2_, and 3-OME_1_) were measured using a Thermo Electron Quantum Discovery Max Triple Quadrupole LC-MS^2^ instrument, with β-glucuronide and sulfate conjugates hydrolyzed [28, 29]. Quantitative analysis was conducted using Thermo Electron Xcalibur proprietary software. Each sample was measured in duplicate and the coefficient of variation was less than 15%. Urinary creatinine concentrations were determined using the Creatinine Colorimetric Assay Kit (Cayman Chemical).

### Statistical analyses

The two-sided Wilcoxon rank sum test or signed rank test was used to compare estrogen or estrogen metabolites levels between two groups (as described in the results). Multiple comparisons were adjusted by calculating the Benjamini-Hochberg FDR [30].

## SUPPLEMENTARY MATERIALS TABLE




