# Supplementary material for: Estrogen metabolism in the human lung: impact of tumorigenesis, smoke, sex and race/ethnicity
Source: Oncotarget. 2017 Nov 1;8(63):106778–89. doi: 10.18632/oncotarget.22269 (PMC5739773; doi:10.18632/oncotarget.22269)
Supplement: Supplementary file 1 [file oncotarget-08-106778-s001.pdf]

Estrogen metabolism in the human lung: impact of tumorigenesis, smoke, sex and race/ethnicity

SUPPLEMENTARY MATERIALS

Supplementary Table 1: Detailed information of each NSCLC patient. See Supplementary_Table_1
